# A More Excellent Way
†"A More Excellent Way." By Constance Howell. (Swan Sonnen schein, Lowrey, and Co.)


**Published:** 1890-02-08

**Authors:** 


**A More Excellent Way.** †

This is a disappointing book, mainly because, in attempting much, the authoress has gone beyond her powers and accomplished little. The seed that in Mrs. Humphrey Ward found such good soil and produced "Robert Elsmere" seems here

† "A More Excellent Way." By Constance Howell. (Swan Sonnenschein, Lowrey, and Co.)

to have fallen by the wayside, and developed into a sickly plant that is little likely to thrive. For, truth to tell, "A More Excellent Way" is very weak. Apparently dissatisfied with nineteenth century Christianity and our latter-day social conditions, Mrs. Howell has, like many another enthusiast, attempted to draw for us architect's plans of a different society, and has failed to give them a semblance of possibility. It seems as if material had been collected for two separate books, and as if, when both had been partly written, for some reason, best known to the author, they had been converted into one. The dove-tailing is bad, and altogether the work lacks finish. "Mrs. Hathaway," in the words of one of the characters, "is a perfect lady, and her son is most intelligent and nice." That is the hero's failing. Rich, good-looking, gentlemanly, an Atheist, and a Socialist, he apparently possesses all that should constitute an ideal hero in these novels with something to preach, and yet he only succeeds in being nice. "Nice" men should be impossible, like Sir Arthur Helps' "tolerable eggs." The minor characters are unobtrusive if somewhat commonplace, but we think Otho Hathaway should be congratulated on his escape from marriage with Miss Evangeline Champneys. If people with religious and socialistic theories to advocate would publish them in the form of sermons or pamphlets, fewer poor novels would be thrust upon an innocent and deserving public.

